# A stitch in time: a cross-sectional survey looking at long lasting insecticide-treated bed net ownership, utilization and attrition in SNNPR, Ethiopia

**DOI:** 10.1186/1475-2875-11-183

**Published:** 2012-06-07

**Authors:** Esey Batisso, Tedila Habte, Gezahegn Tesfaye, Dawit Getachew, Agonafer Tekalegne, Albert Kilian, Betty Mpeka, Caroline Lynch

**Affiliations:** 1 Malaria Consortium, Addis Ababa, Ethiopia; 2 Regional Health Bureau, Southern Nations, Nationalities & Peoples Region, Awassa, Ethiopia; 3 Malaria Consortium, Development House, Leonard Street, London, UK; 4 Malaria Consortium, Upper Naguru, Kampala, Uganda; 5 Department of Infectious and Tropical Diseases, London School of Hygiene and Tropical Medicine, Keppel Street, London, WC1E 7HT, UK

## Abstract

**Background:**

Since 2002/03, an estimated 4.7 million nets have been distributed in the Southern Nations, Nationalities and Peoples Region (SNNPR) among an at risk population of approximately 10 million people. Evidence from the region suggests that large-scale net ownership rapidly increased over a relatively short period of time. However, little is known about how coverage is being maintained given that the last mass distribution was in 2006/2007. This study sought to determine the status of current net ownership, utilization and rate of long lasting insecticide-treated nets (LLIN) loss in the previous three years in the context of planning for future net distribution to try to achieve sustainable universal coverage.

**Methods:**

A total of 750 household respondents were interviewed across malarious, rural kebeles of SNNPR. Households were randomly selected following a two-stage cluster sampling design where kebeles were defined as clusters. Kebeles were chosen using proportional population sampling (PPS), and 25 households within 30 kebeles randomly chosen.

**Results:**

Approximately 67.5% (95%CI: 64.1–70.8) of households currently owned at least one net. An estimated 31.0% (95%CI 27.9–34.4) of all nets owned in the previous three years had been discarded by owners, the majority of whom considered the nets too torn, old or dirty (79.9%: 95%CI 75.8–84.0). Households reported that one-third of nets (33.7%) were less than one year old when they were discarded. The majority (58.8%) of currently owned nets had ‘good’ structural integrity according to a proportionate Hole Index. Nearly two-thirds of households (60.6%) reported using their nets the previous night. The overriding reason for *not* using nets was that they were too torn (45.7%, 95% CI 39.1–50.7). Yet, few households are making repairs to their nets (3.7%, 95% CI: 2.4–5.1).

**Conclusions:**

Results suggest that the life span of nets may be shorter than previously thought, with little maintenance by their owners. With the global move towards malaria elimination it makes sense to aim for sustained high coverage of LLINs. However, in the current economic climate, it also makes sense to hark back to simple tools and messages on the importance of careful net maintenance, which could increase their lifespans.

## Background

Ethiopia aims to eliminate, or achieve near zero transmission of malaria by 2015 [1]. Long lasting insecticide-treated net (LLIN) distribution and community level treatment by health extension workers (HEW) are the cornerstone strategies being used to achieve national goals. The move towards elimination is reflected in the new National Strategic Plan, which outlines a strategy of universal coverage in which all sleeping places are covered with LLINs. Since 2005, it is estimated that more than 20 million LLINs have been distributed to 10 million households in Ethiopia. The 2007 Malaria Indicator Survey showed significant improvements in bed net ownership in malaria risk areas from 3.5% of households owning at least one net in 2005 [2] to 69.0% in 2007 [3]. In the same survey, nearly two-thirds (65.6%) of households owned at least one insecticide-treated mosquito net.

Since 2002/03, an estimated 4.7 million nets have been distributed in the Southern Nations, Nationalities and Peoples Region (SNNPR) among an at risk population of approximately 10 million people. Evidence from the region suggests that large-scale net ownership can be rapidly increased over a relatively short period of time [4]. Various studies in different woredas/districts of the region indicated that between 1999 and 2008 net ownership and utilization rose steadily [4,5] towards Abuja targets [6].

The most recent large scale mass distribution campaigns for LLINs took place in 2007 in SNNPR, which raised the question for Regional Health Bureau teams as to what the current ownership of nets was, given wear and tear and the lifespan of nets. This study sought to determine the status of current net ownership, utilization and rate of LLIN loss in the previous three years in the context of planning for future net distribution to try to achieve sustainable universal coverage.

## Methods

**Figure 1:**
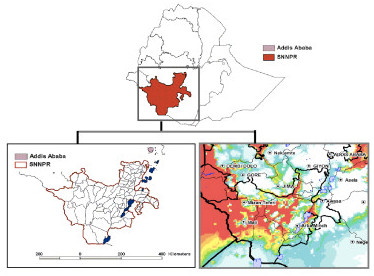
Malaria risk in the study site - SNNPR, Ethiopia.

The study was carried out in December 2009-January 2010, the dry season in this region of Ethiopia, in 14 zones of the Southern Nations, Nationalities and Peoples Regional State (SNNPR), which occupies most of the south-west of Ethiopia. The topography of the region varies from high mountainous areas above 2,500 m altitude to mid-highlands (1,500–2,500 m) down to low rift valley areas of 500–1,500 m. This results in spatially and temporally heterogeneous transmission of malaria in the region. The estimated population of SNNPR is 16 million (extrapolated from Central Statistics Agency, 2007), which represents up to one-fifth of the population of the entire country. The majority of residents at risk of malaria live in the mid-highland areas of the region, characterized by unstable, seasonal transmission (see Figure 1). The Federal Ministry of Health and Regional Health Bureau target LLIN interventions towards “malarious” kebele, which is based on a classification based on history of malaria, rainfall, altitude, proximity to water and other factors.

A two-stage, random sample of 750 household heads were interviewed across malarious, rural kebeles of SNNPR. Malarious kebeles were defined as clusters. Sample size calculations were made on the basis of the primary objective: to determine LLIN utilization. LLIN utilization was estimated at 50% for the study area, on that basis, 384 households needed to be sampled. However, because of the two-stage cluster methodology used to sample households, a design effect of 1.75 was added, giving a total of 672 households to be sampled. An additional 10% was added to the final sample size to adjust for potential loss of data.

A list of all rural malarious kebeles, including their estimated populations and number of households, was obtained from the Regional Health Bureau. A total of 30 SNNPR kebeles to be sampled were chosen using proportional population sampling (PPS), and 25 households within the kebeles chosen by mapping the area and choosing households randomly.

### Ethics

Ethical approval was given from the Regional Health Bureau in SNNPR. In addition, informed written consent was gained from each household head or their equivalent before interviews at the household level. Each interviewed household received a unique identification number consisting of the cluster and the household number. Questionnaires were pre-coded with ID numbers to ensure confidentiality.

### Data collection, management and analysis

A coded, structured questionnaire was adapted from the standard Roll Back Malaria (RBM) Monitoring & Evaluation toolkit. Modifications to the questionnaire were made to align it with the Ethiopian DHS and MIS questionnaires and to add questions regarding LLINs owned in the previous three years. The questionnaire was pre-tested and piloted and adjusted for any issues arising from the piloting. Data was collected in December 2009.

Data was double-entered using Epi info 3.5.1 (Center for Disease Control, Atlanta, USA) and transferred to STATA 11 (Stata Corporation, College Station, Texas, USA) for verification, cleaning and analysis. Descriptive statistical analysis was undertaken to describe the main features of the data in quantitative terms. Simple 2 × 2 tables and chi-squared tests were used to determine the association between variable where relevant. A socioeconomic status index (SES) was developed using principal component analysis (PCA) [7,8].

A proportionate hole index (pHI) was developed so that the integrity of net structure could be categorized. The rationale behind this index is to have proportionality to the average surface of the hole. Holes were counted during the survey for each net in three categories of size named “finger”, “hand” and “head”-size and corresponding to <2cm, 2–10cm and >10cm diameter. Based on previous work in Uganda [9], the hole count was then summarized into a weighted hole index by assuming holes to be functionally squares of sizes 2, 6, and 15 cm on average giving a surface area of 4, 36, and 225 cm^2^ respectively. The ratio of these surface areas was then used as weights to calculate a proportionate hole index (pHI) for each net. At the level of the sampled nets’ physical integrity was then categorized as shown in Table 1.

**Table 1:** Nets were categorised in four ways from the pHI

**LLIN status**	**pHI**
**Good condition**	**<25**
**Fair condition**	**25–174**
**Mediocre condition**	**175–299**
**Poor condition**	**>300**

## Results and discussion

The total number of household respondents was 750 representing a population of 4,706 residents. The average household size was 6.2 (95% CI: 6.1–6.4) occupants. The average number of sleeping spaces per household was 2.5 (95% CI: 2.4–2.6).

Nearly half of heads of households had not received formal education (46.0%: 95% CI 42.2–49.6). However, one-third (30.1%: 95% CI 26.8–39.4) had some primary education (Grades 1–6), while 4.8% (95% CI: 3.2–6.3) had completed primary education.

### Current ownership and utilization of mosquito nets

**Figure 2:**
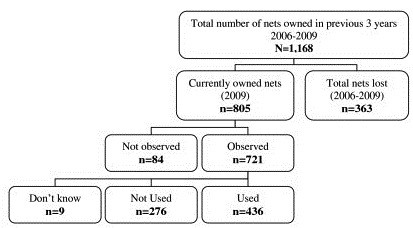
The life of nets in SNNPR 2006–2009.

Households owned a total of 1,168 nets in the three years (2006–2009) prior to the survey (see Figure 1). In total, 470 households reported currently owning 805 nets, of which 721 were observed by interviewers during the survey (see Figure 2). Of the households who owned nets, over one-third of reported that had owned one mosquito net (31.8%: 95% CI 28.3–35.2), half (54.1%: 95% CI 50.5–57.8) owned two nets, 11% (95% CI: 8.7–13.3) had three nets and 3.1% (95%CI: 1.8–4.3) or more than three nets. The average number of nets per net-owning household was 1.86 (95% CI: 1.82–1.92). Approximately 63.6% of households owned any type of mosquito net (95% CI 60.6–67.4), and more than half of households (56.5%: 95% CI 53.1–60.1) owned an LLIN (see Table 2).

**Table 2:** Universal Access Indicators in SNNPR, Ethiopia (2009)

	**n/N**	**%**	**Mean**	**95% CI**
**Number of sleeping places per household**	750	-	2.5	2.4–2.5
**Number of people per household**	750	-	6.2	6.1–6.4
**Mean number of LLIN per household**	750	-	1.9	1.81–1.91
**Households with any type of mosquito net (observed net)**	470/750	67.5	-	60.6–67.4
**Households with at least one LLIN (observed net)**	424/750	56.5	-	53.1–60.1
**Households with net to sleeping space ratio ≥1**	472/750	62.9	-	59.5–66.4
**Households with persons to net ratio of ≤2**	312/750	41.6	-	38.1–45.1
**Percent of nets correctly hung through demonstration**	330/472	69.9	-	65.8–4.1

Over half of all net-owning households (60.5%: 95% CI 56.9–64.1) reported that they had used a net the previous night. Household respondents were asked to demonstrate the correct use of one of their nets of which 97.9% obliged. Among those who demonstrated net use 69.9% (95% CI: 65.7–74.1) hung their nets correctly, that is, hung over the sleeping place, tucked or weighted down under the mattress or by sleeping mat.

The highest proportion of all mosquito nets were used the previous night by the households’ mother, father and youngest child or mother and youngest child together (27.2%: 95% CI 23.9–30.4). On average 9.4% (95% CI 7.3–11.6) were used by children alone. Over one-tenth of households (13.8%: 95% CI 11.3–16.4) reported that the net was used by mother and father alone. Over one-third (38.3%: 95% CI 34.7–41.8) of nets were not used the previous night to the survey. Even though the survey was undertaken in the dry season, most nets were reportedly used throughout the year (75.7%).

**Figure 3:**
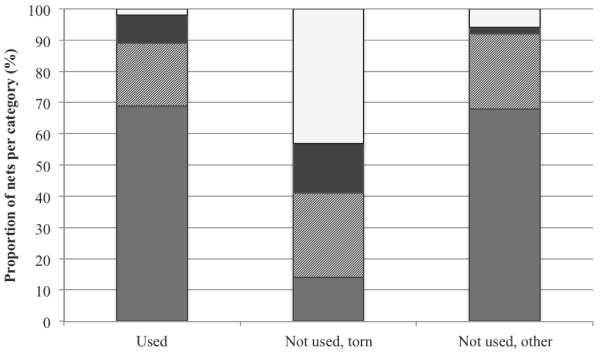
Number of holes in nets reported to be unusable by net-owners (n = 126) compared to other net non-users (n = 150).

The main reason for not using nets was that they were considered too old or torn (45.7%: 95% CI 39.1–50.7). One-fifth of nets (21.7%: 95% CI 16.6–26.2) were considered too dirty to use, while 7.0% (95% CI: 4.0–10.0) of nets were unavailable for use because they were being washed. Interestingly, the physical condition of nets that were considered too torn to use, was broadly similar to those that were used and thus “usable”. Although a large proportion of non-used nets were of “poor” (42.9%) condition, a similar proportion of non-used nets were in “good” (15.6%) or “fair” (25.8%) condition (see Figure 3).

The last mass distribution campaigns took place in late December 2006/early 2007. Nearly one-quarter (23.2%) of all nets were reported to be over 3 years of age. Nearly the same proportion (23.5%) of all nets were reported to be 2–3 years old, 13.9% were 1–2 years old and one-tenth (11.2%). A large proportion of net owners were unsure (28.1%) of what age their nets were, reflecting the difficulty of recalling when nets were received. It is difficult to compare the results from this study with those of most recent surveys carried out in SNNPR because of their patchy geographical coverage [3,5,6,10-14]. Regardless, ownership measured through this survey is lower than those reported in most recent studies [6,13].

### Structural integrity of currently owned nets

The majority (58.8%: 95% CI 55.2–62.4) of observed nets were in “good condition” according to the pHI. A smaller proportion of nets were either in “fair” (22.2%: 95% CI 19.2–25.1) or “mediocre” (7.3%: 95% CI 5.5–9.1) condition and 11% of nets were in “poor” condition. Unsurprisingly, older nets were significantly more likely to have more holes (p < 0.001). However, the majority (70.0%) of nets three years or older were in “good” or “fair” condition. More than likely the ‘fittest’ nets are surviving, and those which are unusable discarded.

Very few households had repaired their nets (3.7%: 95% CI 2.4–5.1), probably because the vast majority of nets were still in “good” or “fair” condition. Of net-owning households who had nets of “mediocre” or “poor” condition on average 34.1% (95% CI: 25.7–42.5) reported that they did not know how to make repairs and the same proportion again reported that they did not realise their net needed repairing.

### Previously owned nets

One-third (32.0%) of nets which households reported owning in the previous three years were thrown away, lost or destroyed. Of the nets discarded in the previous three years (n = 363), most were thrown away because they were considered too old, torn or worn-out (79.9%: 95% CI 75.8–84.0). Some households commented that the nets had finished their chemicals and were burned as a result. Worryingly, one-third of nets (33.7%: 95% CI 28.8–38.5) were less than 12 months old when thrown away. These are relatively high attrition rates for nets, given the current best estimates for net attrition rate of nets with a median three-year survival in the first year of a net is approximately 8%, and 20% and 50% for years 2 and 3 respectively [15].

On average one-third of nets (31.5%: 95% CI 26.6–36.3) were between 12–24 months old when discarded. One-fifth of nets (20.9%: 95% CI 16.9–25.1) were thrown away by households when they were between 24–36 months old, while only 3.5% (95% CI: 1.6–5.4) were older than 36 months. Why were so many nets discarded within 24 months? One possibility is that net wear and tear was greater than previously assumed across Africa.

For this survey, heads of households interviewed were asked whether, and how often in the previous 3 months, they had washed their nets. Most heads of households (75%) stated that they had washed their nets and that on average this took place 3.2 (95% CI: 2.9- 3.5) times per quarter, an annual equivalent of which is nearly 13 times or 38 times over three years, almost double the manufacturers recommends washes. This deviates from the study by Dagne *et al*[11] undertaken in SNNPR who reported that 43.7% of nets had been washed during their lifetime.

Data from previous net ownership and current non-use of nets raises an important issue in terms of net owners understanding of when nets are not usable relative to when protection offered through both insecticide and net integrity is lost. While a net with holes could lead to an annoyingly sleepless night for its owner, there is still a wider public health benefit because of the effect of insecticide on the net. However, convincing a tired net user that this is the case will be difficult if they perceive their net to be ineffective because they are being bitten.

A practical solution would be to provide education on how to maintain nets in either “good” or “fair” condition so that both the owner and community continue to benefit from the use of the net. In SNNPR, the majority of household heads received education from a HEW in the six months prior to this survey. However, the majority of those messages centred on environmental sanitation (44.8%), with mosquito net messages being given in only 36.1% of the time.

**Figure 4:**
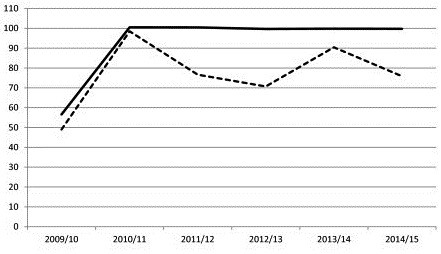
The impact of early loss of nets through attrition on overall net ownership in SNNPR.

If the current coverage estimates and average sleeping spaces in SNNPR were used to plan for net distributions between 2010–2015 then an estimated 9.33 million nets would be required to achieve 100% ownership of nets. However, relatively high net attrition rates, could reduce coverage from 100% to approximately 75% in the first year after a large-scale distribution. This trend would continue and net coverage would not reach 100% until 2015 (see Figure 4). The cost implications would be an additional USD 9.8 million required to keep coverage at 100% in SNNPR with net owners current attitudes towards net effectiveness.

There are a number of limitations to the study. Firstly, it was undertaken during the dry season in SNNPR, during which fewer nets are used than during the rainy season. Questions were asked about seasonal use of nets to try to mitigate this limitation and the majority of household heads reported using nets year-round. Regardless, there could have been issues with recall of net use during rainy seasons. The same issue of recall applies to questions regarding the ages of currently owned nets, and previously owned nets.

Secondly, there are major challenges in trying to measure the structural integrity of nets. In this study a proportionate Hole Index recently suggested by the LLIN durability work stream of the RBM Vector Control Working Group (A.Kilian, *pers comm*). There are significant challenges to using this index: firstly, there are difficulties in taking good quality measurements of hole sizes in a setting where a large number of nets are being evaluated, and secondly, it is difficult to categorize the condition of nets (i.e. “good”, “fair” etc) in a meaningful way. In reality, the definition of a ‘failed’ or ‘poor condition’ net is still very much open to question. The amount of insecticide remaining on nets was not measured during the survey, so it is not possible to assume that the amount of insecticide remaining on older nets was sufficient or not. In addition, evidence has not yet been established as to the association between pHI and net effectiveness.

## Conclusions

A two-stage cross-sectional survey was undertaken in December 2009 in SNNPR, Ethiopia in order to determine current LLIN ownership and rates of net utilization, rate of loss of nets and maintenance. Results show that 67.5% of households owned at least one net of any type. One-quarter of households owned two nets and fewer than 5% owned three or more. Of those nets owned, 60.5% were used the previous night, 69.9% of net owners who agreed to demonstrate their use did so correctly.

The structural integrity of the current net population was relatively good. However, data from previous nets owned and non-used nets indicates that the net attrition rate may be higher than previously thought. Nets are frequently washed, but rarely repaired by their owners. An adjustment to the communication strategy in SNNPR so that HEWs give out key messages on how often to wash nets and an emphasis on their maintenance, as well as the usefulness of nets even with holes, could go some way towards ensuring an extended life span of the net population. Further research to understand what households define as an ‘unusable’ net and what their actions normally are as a nets physical integrity begins to wane is needed to further bolster communication campaigns.

With the global move towards malaria elimination it makes sense to increase coverage with sustained high coverage of LLINs. However, in the current economic climate, it also makes sense to hark back to simple tools and messages on the repair and washing of nets, which could serve to increase their life spans. A stitch in time could, ultimately, save a great deal of money.

## Competing interests

The authors declare that they have no competing interests.

## Authors’ contributions

CAL designed, analysed survey results and drafted the manuscript. EB designed and implemented survey and inputted into manuscript drafts. TH designed and implemented the survey. AT inputted into the design, training and implementation of the survey as well as manuscript review. DG inputted into the design, training and implementation of the survey. GT inputted into the design, analysis and manuscript reviews. AK inputted into the design and analysis of survey results as well as reviewing the manuscript. All authors read and approved the final manuscript.
